# Three-Wave Longitudinal Survey on the Relationship between Neuroticism and Depressive Symptoms of First-Year College Students: Addictive Use of Social Media as a Moderated Mediator

**DOI:** 10.3390/ijerph17176074

**Published:** 2020-08-20

**Authors:** Weiqi Mu, Dongyun Zhu, Yanhong Wang, Fugui Li, Liyuan Ye, Kexin Wang, Mingjie Zhou

**Affiliations:** 1 CAS Key Laboratory of Mental Health, Institute of Psychology, Chinese Academy of Sciences, Beijing 100101, China; muwq@psych.ac.cn (W.M.); aschenpeter@126.com (D.Z.); lifg@psych.ac.cn (F.L.); yeliyuan06@gmail.com (L.Y.); 2 Department of Psychology, University of Chinese Academy of Sciences, Beijing 100049, China; 3 Mental Health Counseling Center, Yang’ En University, 362014 Quanzhou, China; wangyh@yeu.edu.cn; 4 College of Media and International Culture, Zhejiang University, Hangzhou 310007, China; wangkexin0809@zju.edu.cn

**Keywords:** depressive symptoms, neuroticism, addictive use of social media, psychological resilience, moderated mediation

## Abstract

First-year college students’ adaptation problems and related mental health have attracted researchers’ attention. The current research focuses on the depressive symptoms of first-year college students and aims to explore the relationship between the neuroticism trait and depressive symptoms, the mediating effect of addictive use of social media, and the moderating effect of psychological resilience. Three-wave longitudinal data from 1128 first-year students at a university in Fujian Province, China, were collected within three months of their enrollment. PROCESS macro for SPSS with bootstrapping was used to test the model. Results showed that the prevalence of moderate to severe severity of depressive symptoms in first-year students was 10.28% (T1) and 11.17% (T3). Addictive use of social media (T2) plays a moderated mediator role in the relationship between neuroticism (T1) and depressive symptoms (T3) of first-year students. Specifically, a low neuroticism individual does not necessarily have a less addictive use of social media. Psychological resilience (T1) moderated the above mediation. Implications for research and practice are discussed.

## 1. Introduction

While college presents many students with opportunities for personal growth and enhancement, some students find the demands of college adjustment exceed their coping resources [1]. New college students begin their transition from adolescence to adulthood, and many of them have difficulty adapting to college life. The worse the adaptation of first-year students, the higher the risk of psychological disturbance [2,3]. Previous studies have shown that there is a significant correlation between depression symptoms and suicide ideation of college students [4,5]. Therefore, the mental health of first-year college students, especially depressive symptoms, deserves our attention. Many researches have demonstrated the closing association between personality traits and depression, especially the role of high neuroticism in developing depression [6,7,8,9]. Meanwhile, addictive use of social media has become an area of increasing research interest. A large national survey in Norway has explored that being a student and young is associated with a high risk of addictive use of social media [10]. Addictive use of social media is associated with both neuroticism traits and depression [11,12]. Therefore, in this study, we aim to provide insight into how, and under what conditions, neuroticism leads to a higher level of depressive symptoms from the perspective of the addictive use of social media.

### 1.1. The Relationship between Neuroticism and Depressive Symptoms

The “Big Five” personality model (openness, conscientiousness, extraversion, agreeableness, neuroticism), as the most influential personality theoretical model in the world, has been widely studied by many psychological researchers in the past half-century and has been proved to have the relative consistency and stability across different samples, raters, languages, and cultures [13]. The personality trait of neuroticism refers to relatively stable tendencies to respond with negative emotions to threat, frustration, or loss [14]. Negative emotionality is the underpinning of neuroticism [15,16]. Individual neuroticism levels lead to differences in perception, memory, affection, and behavior during emotional stimuli and negative events, which explains why some individuals may be better at regulating their emotional state than others [17]. Neuroticism is significantly correlated with higher scores in depressive symptoms and appears to be the most powerful predictor of depression [18,19].

### 1.2. The Relationship between Addictive Use of Social Media and Depressive Symptoms

With the development of computers and networks, people’s communication mode has gradually shifted from reality to networks. According to the Pew Research Center, 72% of American adults use social media as of February 2019, including 90% of young people aged 18–29 [20]. By March 2020, the number of Internet users in China has reached 904 million, of which 99.3% are mobile Internet users. In terms of occupation, student users account for 26.9% for the most substantial proportion. Among all kinds of social applications, WeChat has the highest usage rate, reaching 85.1% [21]. Some studies have indicated a positive relationship between social media and mental health [22,23]. However, many others hold the opposite opinion, which shows that psychological distress is related to maladaptive use of both the Internet and the mobile phone [24,25,26]. Addictive use of social media (similar concepts include “social media use disorder”, “social network overuse”, “pathological social network use”, “social network addiction”) has a negative impact on adolescents’ physical and mental development [27]. Overuse of Facebook can lead to depression, according to a report from the American Academy of Pediatrics [28]. Many studies have indicated that adolescents’ problematic social network usage is positively correlated with depressive symptoms [29,30]. Therefore, the addictive use of social media of first-year students is worthy of attention.

### 1.3. Addictive Use of Social Media as a Mediator in the Relationship between Neuroticism Trait and Depressive Symptoms

Furthermore, previous studies showed that Facebook addiction was positively related to neuroticism [31,32]. Individuals with high neuroticism tend to use social media more frequently [33]. Introverted and neurotic people locate their “real me” on the Internet, while extroverts and non-neurotic people locate their “real me” through traditional social interaction [34]. Young adults high in neuroticism are inclined to present their ideal and false self on Facebook to a greater extent [35]. High-neurotic individuals may be more willing to seek emotional satisfaction and stress release through mobile phones to avoid the real world [36]. Therefore, we speculate that first-year college students with high neuroticism are more likely to be addicted to social media in the face of adaptive pressure, which will further aggravate their risk of depression. In other words, the addictive use of social media plays a mediating role between neuroticism and depressive symptoms.

### 1.4. Moderation of Psychological Resilience

Human responses to stress and trauma vary widely. Resilience is an interactive concept that refers to a relative resistance to environmental risk experiences or the overcoming of stress or adversity [37]. Psychological resilience is a protective factor of individuals in time of stress [38] and can increase the odds of not being depressed [39]. Among all independent variables, resilience is the most effective at predicting first-year university students’ ability to adjust to university life [40]. At the same time, psychological resilience is a critical factor in protecting adolescents from internet addiction [41]. However, considering that neuroticism is the risk factor of individuals’ addictive use of social media, while psychological resilience may be the protective factor for addictive use of social media, combined with the theory of the buffering effect of psychological resilience [42], this study assumes that psychological resilience may play a moderating role between neuroticism and addictive use of social media, namely psychological resilience may reduce the risk of addictive use of social media of highly neurotic individuals.

**Figure 1:**
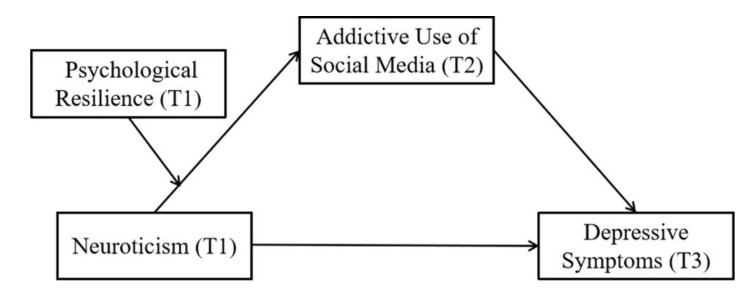
Moderated mediation model of the present research.

To sum up, based on the relationship between neuroticism and depressive symptoms, this study hypothesized a moderated mediation model (see Figure 1). We aimed to provide insights into how (the mediation role of addictive use of social media), and under what conditions (the moderator role of psychological resilience), neuroticism leads to a higher level of depressive symptoms.

## 2. Materials and Methods

### 2.1. Participants and Procedures

Data were collected at three different periods among 1428 students who began their freshman year at a university in China, in 2017. The majors of these students include Law, Financial Management, Accounting, Electronic Information Engineering, Journalism, Advertising, Chinese Language and Literature, Marketing, Logistics Management, Economics, Network Engineering, English Language and Literature, Industrial Engineering, Business Administration, International Economics and Trade, Finance, Computer Science and Technology, and Electrical Engineering and Automation. The participation was anonymous and voluntary, based on the invitation from a course teacher responsible for the mental health education of this university. The teacher conducted the survey in each class on the spot with the participants’ informed consent. The study protocol was approved by the institutional review board: Ethics Committee of Institute of Psychology, Chinese Academy of Sciences. Three waves of data were collected within two months after enrollment, considering that the first semester or even the first few weeks of college may be especially crucial in terms of student adaptation [43]. In wave 1 (October 2017), a total of 1428 students’ demographic variables (T1), neuroticism (T1), psychological resilience (T1) and depressive symptoms (T1) were tested, and 1350 valid questionnaires were recovered, with an effective recovery rate of 94.54%. In wave 2 (November 2017), the data of 1423 first-year students’ addictive use of social media (T2) were collected, and 1262 valid questionnaires were recovered, with an effective recovery rate of 88.69%. In wave 3 (December 2017), 1425 first-year students’ depressive symptoms (T3) were measured, and 1271 valid questionnaires were recovered, with an effective recovery rate of 89.19%.

Finally, the data of 1128 participants who were all valid in the three waves were included in the analysis, after eliminating the samples with obvious regularity, a large number of blank answers or those who only participated in one or two surveys. The range of participants’s age was 17–22 years old (M = 18.74; SD = 0.48). Among them, 731 (64.80%) were female, 323 (28.63%) were the only child in their family, and 309 (27.40%) had their family in the city where the university is located.

### 2.2. Measurement

#### 2.2.1. Neuroticism

Neuroticism was measured by the neuroticism subscale of the Ten-Item Personality Inventory (TIPI) [44], which contains two 7-point Likert items (“Anxious, easily upset.” and “Calm, emotionally stable.”; 1 = Disagree strongly, 7 = Agree strongly). The second item above was scored in reverse. The correlation between neuroticism in the TIPI scale and that in BFI-44 is 0.81 [44]. The Chinese version of TIPI has good psychometric qualities in the Chinese sample [45].

#### 2.2.2. Depressive Symptoms

The Patient Health Questionnaire (PHQ-9) [46] was used to assess the severity of depressive symptoms. The scale contains nine 4-point Likert items (0 = Not at all, 3 = Nearly every day). An example of the items is “Over the last two weeks, how often have you been bothered by any of the following problems? Little interest or pleasure in doing things”. In the present study, we use ten as the cut-off score [47,48]. Students with a total score of not lower than ten on PHQ-9 were judged as moderate to severe depressive symptoms severity. The Chinese version of PHQ-9 held good psychometric qualities in the Chinese sample [49]. Cronbach’s alpha was 0.84 in this study.

#### 2.2.3. Addictive Use of Social Media

The measurement of addictive use of social media referred to the FB addiction dimension in the Psycho-Social Aspect of Facebook Use Scale (PSAFU) [50], which includes four 5-point Likert items (1 = completely inconsistent; 5 = completely consistent). By replacing “Facebook” with “SNSs,” we revised the scale to be more suitable for Chinese college students. An example of the items is “Some people from my surrounding have told me that I spend too much time on the SNSs.” In this study, Cronbach’s alpha was 0.69.

#### 2.2.4. Psychological Resilience

Psychological resilience was measured by the 10-item Connor–Davidson Resilience Scale (10-item CD-RISC) [51], which contains ten 5-point Likert items (1 = never; 5 = always). An example of the items is “Able to adapt to change.” The higher the average score, the higher the psychological resilience level of the participants. The Chinese version of the 10-item CD-RISC held good psychometric properties and is applicable for Chinese people [52]. In this study, Cronbach’s alpha was 0.90.

### 2.3. Analytical Strategy

PROCESS macro for SPSS with bootstrapping (95% CI, 5000 samples) was used to test the moderated mediation model [53]. Controlled variables from wave 1 were added as covariates in the model, including gender (1 = Male, 0 = Female), age, the only child or not in the family (1 = Yes, 0 = No), location of the family (1 = In the city where the university is located, 0 = Outside the city where the university is located) and the baseline level of depressive symptoms.

## 3. Results

### 3.1. Prevalence of Moderate to Severe Severity of Depressive Symptoms

Among the 1128 students, the prevalence of moderate to severe severity of depressive symptoms was 10.28% in the first survey. There were 35 cases with moderate to severe severity of depressive symptoms (8.82%) among 397 male students and 81 (11.08%) among 731 female students. There was no statistically significant difference in the prevalence of moderate to severe severity of depressive symptoms between male and female students (χ^2^ = 1.43, *p* = 0.232) in the first survey.

In the third survey, the prevalence of moderate to severe severity of depressive symptoms was 11.17% among the 1128 students. There were 47 cases with moderate to severe severity of depressive symptoms (11.84%) among 397 male students and 79 (10.81%) among 731 female students. There was no statistically significant difference in the prevalence of moderate to severe severity of depressive symptoms between male and female students (χ^2^ = 0.276, *p* = 0.599) in the third survey.

Eighty-three students (7.36%) who did not have moderate to severe depressive symptoms in the first survey turned to have in the third survey. On the other hand, 73 students of the total 1128 students (6.47%) who had moderate to severe depressive symptoms in the first survey turned not to have in the third survey.

### 3.2. Descriptive Statistics and Correlation Analysis

Descriptive statistics and correlation analysis results were shown in Table 1. Neuroticism (T1) was positively correlated with depressive symptoms (T1). Addictive use of social media (T2) and depressive symptoms (T3), addictive use of social media (T2) was positively correlated with depressive symptoms (T1) and depressive symptoms (T3). Psychological resilience was negatively correlated with neuroticism (T1), depressive symptoms (T1), addictive use of social media (T2) and depressive symptoms (T3).

### 3.3. Relationship Between Neuroticism and Sepressive Symptoms: Test of a Moderated Mediation Model

The results of polynomial regression analysis were shown in Table 2. After controlling for gender, age, the only child or not in family, location of family, and depressive symptoms level (T1), neuroticism (T1) positively predicted depressive symptoms (T3) (*B* = 0.03, *SE* = 0.01, *t* = 2.71, *p* = 0.007) significantly. Neuroticism (T1) had a significant positive effect on levels of addictive use of social media (T2) (*B* = 0.05, *SE* = 0.02, *t* = 2.34, *p* = 0.02). Addictive use of social media (T2) positively predicted depressive symptoms (T3) (*B* = 0.10, *SE* = 0.01, *t* = 7.64, *p* < 0.001) significantly. After controlling for addictive use of social media (T2), neuroticism (T1) still positively predicted depressive symptoms (T3) (*B* = 0.02, *SE* = 0.01, *t* = 2, *p* = 0.045) significantly. The Bootstrap test using Model 4 of PROCESS showed that addictive use of social media (T2) played a significant mediating role between neuroticism (T1) and depressive symptoms (T3) (95% Boot CI = (0.002, 0.013)).

**Figure 2:**
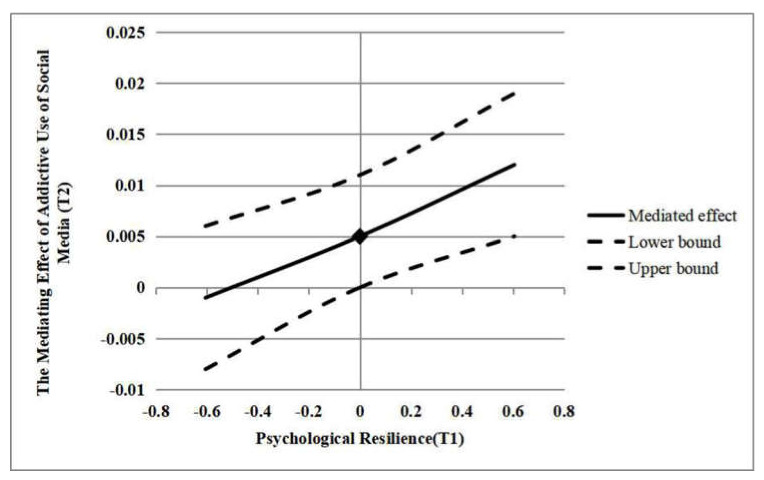
The mediating effect of addictive use of social media (T2) at different levels of psychological resilience (T1).

To examine the moderation effect of psychological resilience (T1) in this mediation model, we applied Model 7 in PROCESS macro to do the Bootstrap test. Neuroticism (T1) and psychological resilience (T1) were centralized before constructing the interaction term. The results showed that the mediating effect was significantly moderated by psychological resilience (T1), indicating that the moderated mediation model was established (95% Boot CI = (0.003, 0.019)). Specifically, the mediating effect was not significant at low level of psychological resilience (−1 SD; 95% Boot CI = (−0.008, 0.006)), while was significant at medium level (95% Boot CI = (0.000, 0.011)) and high level of psychological resilience (+1 SD; 95% Boot CI = (0.005, 0.019)). The mediating effect of addictive use of social media (T2) at different levels of psychological resilience (T1) was shown in Figure 2.

**Figure 3:**
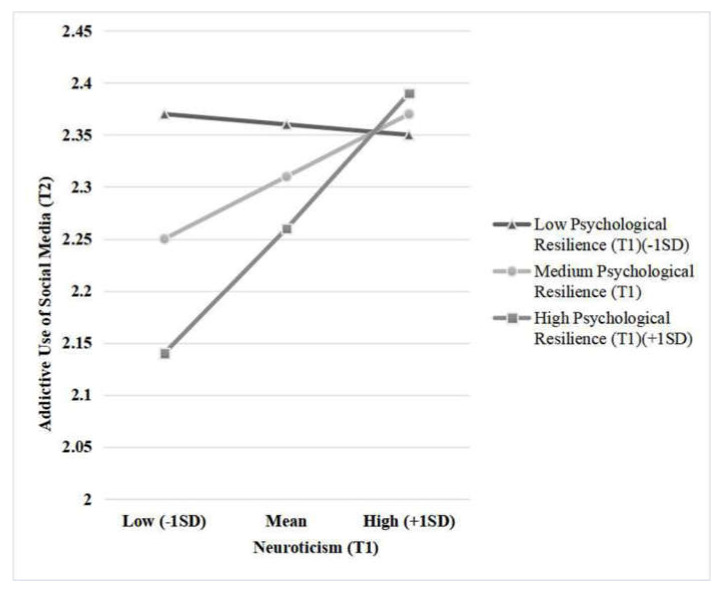
Simple slope test of the interaction between neuroticism (T1) and psychological resilience (T1) on the addictive use of social media (T2).

Meanwhile, the neuroticism (T1) × psychological resilience (T1) interaction significantly predicted addictive use of social media (T2) (*B* = 0.10, *SE* = 0.03, *t* = 3.63, *p* < 0.001; see Figure 3). The influence of neuroticism (T1) on addictive use of social media (T2) was not significant when the level of psychological resilience (T1) is low (*B* = −0.01, *SE* = 0.03, *t* = −0.31, *p* = 0.757) and was significant when psychological resilience (T1) is at medium (*B* = 0.05, *SE* = 0.02, *t* = 2.34, *p* = 0.02) and high level (*B* = 0.11, *SE* = 0.03, *t* = 4.23, *p* < 0.001).

## 4. Discussion

In this study, the relationship between neuroticism and depressive symptoms of first-year students was investigated by longitudinal tracking. The mediating and moderating effects of addictive use of social media and psychological resilience were explored, and a moderated mediation model was constructed. The results help to understand the two critical issues of how and under which conditions the neuroticism of first-year college students affect their depressive symptoms.

### 4.1. Prevalence of Moderate to Severe Severity of Depressive Symptoms

Among the 1128 students, the prevalence of moderate to severe severity of depressive symptoms was 10.28% in the first survey, and 11.17% in the third survey with the cut-off score no less than 10 of PHQ-9 scores. Of the 397 male students, 35 cases had moderate to severe severity of depressive symptoms (8.82%) in the first survey and 47 (11.84%) in the third survey. Of the 731 female students, 81 cases had moderate to severe severity of depressive symptoms (11.08%) in the first survey and 79 (10.81%) in the third survey. Moreover, 83 students (7.36%) who did not have moderate to severe severity of depressive symptoms when they enrolled in the college turned to do two months later. However, previous studies showed that the prevalence of depression under the same standard ranged from 4.23% to 7.00% in the general Chinese population [49,54], which is 9.56% in males and 5.60% in females [49]. The results suggest that first-year students have a higher prevalence of moderate to severe severity of depressive symptoms than that in the general population, representing it is necessary to pay attention to and research the adaptation problems and depressive symptoms of first-year students, considering the higher risk of depression for the students with poor school adjustment [3].

### 4.2. Mediation of Addictive Use of Social Media

This study shows that first-year college students’ neuroticism can significantly predict depressive symptoms two months later. High neuroticism people usually show more emotion dysregulation and psychological inflexibility, which can also explain why individuals with high neuroticism are more prone to depression [9]. This study also found that neuroticism can significantly predict the addictive use of social media, indicating that the higher the level of neuroticism, the more likely an addiction to social media, which is consistent with the previous research results [33,55]. Meanwhile, the addictive use of social media can significantly predict depressive symptoms. The higher the intensity of first-year students’ addictive use of social media, the higher the risk of depression, which is in line with previous researches [28,29,56].

This study confirmed that the addictive use of social media plays a significant mediating role in the influencing process of neuroticism on depressive symptoms. This paper further explains the mechanism of neuroticism’s influence on depressive symptoms; that is, neuroticism affects the depressive symptoms level of first-year students directly and indirectly through addictive use of social media, which serves as a bridge between neuroticism and depressive symptoms.

### 4.3. Moderation of Psychological Resilience

Psychological resilience plays a moderating role in the relationship between neuroticism and the addictive use of social media. The improvement of psychological resilience does not reduce the risk of addictive social media use of high neurotic individuals. However, it protects the low neuroticism ones. Namely, with the improvement of psychological resilience, low neuroticism individuals reduce their addictive use of social media significantly. This also suggests that in addition to the buffering effect (reducing the risk of high-risk individuals), there is also an increasing effect (making low-risk individuals perform better) of psychological resilience, which echoes the current research results [57]. Our results show that low neurotic individuals likewise have a risk of addictive use of social media if their psychological resilience level is not high.

At the same time, this study has constructed a moderated mediation model to investigate the moderating role of resilience in the mediating process of “neuroticism -> addictive use of social media -> depressive symptoms”. The results show that neuroticism directly affects the depressive symptoms level of individuals with low resilience. With the improvement of resilience, neuroticism impacts the level of depressive symptoms through the mediating effect of addictive use of social media. Therefore, first-year college students with high psychological resilience, regardless of their neuroticism, can avoid depression and promote their adaptation to college life from the perspective of intervention in the addictive use of social media. However, this approach is not appropriate with low resilience, and other interventions should be sought. For example, when both neuroticism and resilience are low, the latter can be improved by the optimal level of participation in sports activities according to gender and age [58].

## 5. Implication and Limitation

### 5.1. Implication

Theoretically, the present study implied that low neuroticism people with low psychological resilience might also have a risk of addictive use of social media. Second, psychological resilience acts not as a buffer, but as an increasing effect, making people with low neuroticism less likely to be addicted to social media. Third, the mediating effect of addictive use of social media is different in people with different psychological resilience. That is, the mediating effect was not significant at the low level of psychological resilience, but was significant at the medium and high level of psychological resilience. These results provide a more sophisticated knowledge of understanding the depressive symptoms of first-year college students.

Practically, the present study provides insights into how and under what conditions, neuroticism leads to a higher level of depressive symptoms, which enables several practical implications for reducing the risk of depression of first-year college students with adaptation problems: (a) after controlling the baseline level of depressive symptoms, neuroticism and addictive use of social media are all risk factors of first-year college students’ depression. Therefore, research on the personality traits of first-year college students and dynamic evaluation of their social media behaviors can be undergone after enrollment to predict their depressive symptoms two months after effectively. (b) More accurate classification guidance can be offered based on differences in first-year college students’ personality characteristics and resilience. On the one hand, the possibility of the addictive use of social media of low neuroticism individuals can be reduced by improving their psychological resilience according to the moderating effect. On the other hand, based on the results of the mediating effect, individuals with high resilience need attention to avoid the addictive use of social media to better adapt to college life.

### 5.2. Limitation

Although some valuable results have been obtained in this study, there are still some deficiencies. First, neuroticism in this study involves a relatively broad personality construct without subdividing different aspects. In future research, the neuroticism personality dimension can be subdivided to distinguish the impact of different aspects on the relationship between addictive use of social media and depressive symptoms. Second, this study used longitudinal data to analyze and provided evidence to support the hypothesized path from the addictive use of social media to depressive symptoms. However, some studies pointed out that depression was a critical antecedent variable of social media addiction [59,60]. Future research can start from this perspective to explore the more abundant dyadic relationship between the two.

## 6. Conclusions

The current research focuses on the depressive symptoms of first-year college students. It aims to provide insights into how (the mediation role of addictive use of social media), and under what conditions (the moderator role of psychological resilience), neuroticism leads to a higher level of depressive symptoms. The most obvious findings to emerge from this study are that low neuroticism people with low psychological resilience might also have a risk of addictive use of social media, psychological resilience acts as an increasing effect decreasing the risk of low neuroticism individual’s addictive use of social media, and addictive use of social media plays the mediating effect only with medium and high psychological resilience. The insights gained from this study may help understand the depressive symptoms of first-year college students and provide intervention guidance for the adaptation problems.

## Figures and Tables

**Table 1:** Means, standard deviations, and correlations among research variables.

Research Variables	M	SD	1	2	3	4	5	6	7	8
1.Gender (male = 1 female = 0)										
2.Age	18.74	0.83	−0.02							
3.The Only Child or Not			0.239 ***	−0.134 ***						
4.Location of Family			−0.045	0.016	−0.032					
5.Depressive symptoms (T1)	0.62	0.38	−0.080 **	0.011	−0.066 *	0.043				
6.Neuroticism (T1)	3.60	1.12	−0.111 ***	−0.012	−0.029	−0.02	0.411 ***			
7.Psychological Resilience (T1)	3.51	0.61	0.180 ***	0.041	0.05	−0.022	−0.355 ***	−0.415 ***		
8.Addictive Use of Social Media (T2)	2.28	0.74	−0.014	0.009	−0.001	−0.022	0.214 ***	0.177 ***	−0.148 ***	
9.Depressive symptoms (T3)	0.67	0.39	−0.022	−0.04	−0.059 *	0.004	0.507 ***	0.270 ***	−0.239 ***	0.300 ***

Note: * *p* < 0.05; ** *p* < 0.01; *** *p* < 0.01.

**Table 2:** Multiple regression results of the moderated mediation model.

Independent Variable	*B*	*SE*	*t*	*p*	*R^2^*	*F*
Dependent Variable: Depressive symptoms (T3)						
Gender	0.03	0.02	1.23	0.221	0.27	67.57 ***
Age	−0.02	0.01	−1.92	0.056		
The Only Child or Not	−0.03	0.02	−1.53	0.127		
Location of Family	−0.01	0.02	−0.53	0.599		
Depressive symptoms (T1)	0.48	0.03	16.91	<0.001		
Neuroticism (T1)	0.03	0.01	2.71	0.007		
Dependent Variable: Addictive Use of Social Media (T2)						
Gender	0.03	0.05	0.66	0.509	0.07	10.55 ***
Age	0.01	0.03	0.26	0.791		
The Only Child or Not	0.02	0.05	0.39	0.700		
Location of Family	−0.04	0.05	−0.92	0.358		
Depressive symptoms (T1)	0.33	0.06	5.27	<0.001		
Neuroticism (T1)	0.05	0.02	2.34	0.020		
Psychological Resilience (T1)	−0.08	0.04	−1.99	0.047		
Neuroticism (T1) × Psychological Resilience (T1)	0.10	0.03	3.63	<0.001		
Dependent Variable: Depressive symptoms (T3)						
Gender	0.03	0.02	1.20	0.230	0.30	69.21 ***
Age	−0.02	0.01	−2.05	0.041		
The Only Child or Not	−0.04	0.02	−1.66	0.098		
Location of Family	−0.01	0.02	−0.33	0.741		
Depressive symptoms (T1)	0.45	0.03	15.89	<0.001		
Neuroticism (T1)	0.02	0.01	2.00	0.045		
Addictive Use of Social media (T2)	0.10	0.01	7.64	<0.001		

Note: *** *p* < 0.001; *B*—unstandardized regression weight; *SE*—standard error for the unstandardized regression weight; *t*—*t*-test statistic; *F*—*F*-test statistic.
